# Methods of applying the 1994 case definition of chronic fatigue syndrome – impact on classification and observed illness characteristics

**DOI:** 10.1186/s12963-016-0077-1

**Published:** 2016-03-12

**Authors:** E. R. Unger, J.-M. S. Lin, H. Tian, B. M. Gurbaxani, R. S. Boneva, J. F. Jones

**Affiliations:** Division of High-Consequence Pathogens and Pathology, National Center for Emerging and Zoonotic Infections, Centers for Disease Control and Prevention, 1600 Clifton Road, MS G41, Atlanta, GA 30329 USA

**Keywords:** Chronic fatigue syndrome, Case definition, Surveillance methods

## Abstract

**Background:**

Multiple case definitions are in use to identify chronic fatigue syndrome (CFS). Even when using the same definition, methods used to apply definitional criteria may affect results. The Centers for Disease Control and Prevention (CDC) conducted two population-based studies estimating CFS prevalence using the 1994 case definition; one relied on direct questions for criteria of fatigue, functional impairment and symptoms (1997 Wichita; Method 1), and the other used subscale score thresholds of standardized questionnaires for criteria (2004 Georgia; Method 2). Compared to previous reports the 2004 CFS prevalence estimate was higher, raising questions about whether changes in the method of operationalizing affected this and illness characteristics.

**Methods:**

The follow-up of the Georgia cohort allowed direct comparison of both methods of applying the 1994 case definition. Of 1961 participants (53 % of eligible) who completed the detailed telephone interview, 919 (47 %) were eligible for and 751 (81 %) underwent clinical evaluation including medical/psychiatric evaluations. Data from the 499 individuals with complete data and without exclusionary conditions was available for this analysis.

**Results:**

A total of 86 participants were classified as CFS by one or both methods; 44 cases identified by both methods, 15 only identified by Method 1, and 27 only identified by Method 2 (Kappa 0.63; 95 % confidence interval [CI]: 0.53, 0.73 and concordance 91.59 %). The CFS group identified by both methods were more fatigued, had worse functioning, and more symptoms than those identified by only one method. Moderate to severe depression was noted in only one individual who was classified as CFS by both methods. When comparing the CFS groups identified by only one method, those only identified by Method 2 were either similar to or more severely affected in fatigue, function, and symptoms than those only identified by Method 1.

**Conclusions:**

The two methods demonstrated substantial concordance. While Method 2 classified more participants as CFS, there was no indication that they were less severely ill or more depressed. The classification differences do not fully explain the prevalence increase noted in the 2004 Georgia study. Use of standardized instruments for the major CFS domains provides advantages for disease stratification and comparing CFS patients to other illnesses.

## Background

Chronic fatigue syndrome (CFS) is a debilitating multi-system illness that compromises occupational, educational, social, or personal activities and is accompanied by fatigue persisting longer than 6 months, as well as a variety of symptoms that may include significant collapse or relapse after exertion (post-exertional malaise), sleep problems, cognitive impairment, dizziness, muscle aches and pains, tender lymph nodes, and headaches. Many case definitions for CFS, as well as for myalgic encephalomyelitis (ME) or ME/CFS, have been proposed and debated in the literature. Those in use include the 1994 case definition [1], the 2003 Canadian case definition [2], the 2010 revised Canadian case definition [3], and the 2011 International Consensus Criteria [4]. Concerns about case definitions used for epidemiologic studies, clinical diagnosis, and research are not unique to CFS, and in fact are common in such diverse illnesses as acute coronary heart disease [5], chronic kidney disease [6], interstitial cystitis [7], periodontitis [8] and toxic shock syndrome [9], to give a few examples.

While surveys indicate that healthcare providers are aware of ME/CFS [10], other reports document the difficulties that physicians have in recognizing an illness that lacks a diagnostic test, as well as the delays and frustrations that patients experience in being diagnosed [11]. With the aim of improved clinical care for ME/CFS, the Institute of Medicine (IOM) recently conducted an in-depth review of the evidence for diagnostic criteria for ME/CFS, considering input from patients as well as the physicians and advocates caring for them. Their report confirms the serious nature of this illness and provides guidance on clinical criteria for ME/CFS to make it easier for clinicians to recognize and diagnose patients in a timely manner (http://www.iom.edu/Reports/2015/ME-CFS.aspx). In recognition of the many gaps in knowledge about this illness and need for more research, the IOM report further recommends reexamining diagnostic criteria in no more than 5 years.

Research on nearly every aspect of ME/CFS such as prevalence of illness, risk factors, disease course, etiology, and response to therapy requires studying well-defined patients or patient subgroups. Case definitions are used to identify patients but have limitations in their ability to accurately and reproducibly classify patients. In addition, variations in study methods extend beyond the case definition. The study population (e.g., clinic versus community), method of recruitment and screening, extent of medical and psychiatric evaluation to rule out other illness, matching criteria for case–control comparisons, and questionnaires/instruments used to ascertain information about participants’ health all have the potential to affect results [12–16]. For chronic illnesses such as ME/CFS, duration of illness, medications, and co-morbid conditions all contribute to heterogeneity. A recent publication suggested that developing consensus on data elements about CFS patients and their illness to be included in research publications could help investigators compare findings across different studies [17]. The case definition is clearly one of these elements, but it is insufficient to simply state which case definition was used without describing how it was operationalized.

CDC has conducted two population-based longitudinal studies of CFS [18, 19]; both used random-digit-dialing to select and survey households, included clinical and laboratory testing to identify exclusionary conditions, and based classification on the 1994 case definition [1]. However, there were two significant differences in the studies: 1) the screening criteria for eligibility to attend the clinical evaluation (both household and individual screening interviews and eligibility criteria), and 2) the method of applying the case definition (questionnaires and case definition algorithms). These differences are highlighted briefly, omitting details that can be found in the original reports [18, 19]. The longitudinal study in Sedgewick County Kansas, initiated in 1997 (1997 Wichita), required respondents to endorse fatigue, and identified symptoms required for the case definition by asking respondents whether or not they experienced each. From this longitudinal study, we observed that a substantial portion of subjects meeting CFS criteria during follow-up were not incident cases, but prevalent cases that were not identified in initial surveillance cycles. This largely occurred because fatigue was used as the sole screening criteria during the household informant interview. Households with an individual whose illness was not perceived as involving fatigue during the interview, either by the household informant or themselves, were not selected or evaluated at the clinic. Therefore, when we initiated the Georgia surveillance study in 2004 (2004 Georgia), we expanded the screening interviews to the four major core symptoms of the syndrome: fatigue, cognitive impairment, unrefreshing sleep, and pain. Furthermore, following published recommendations, standardized questionnaires were used to measure the three domains of illness required by the 1994 case definition (fatigue, functional impairment, and symptoms) [14, 20].

These changes in the method of applying the 1994 case definition resulted in concerns about whether the cases identified in the Georgia study truly had CFS, or if the expanded screening criteria and reliance on questionnaires resulted in misclassification of persons with psychiatric co-morbidities or inclusion of relatively mild or non-specific fatiguing illness [21]. We used the data from the follow-up (GA-T1) of the baseline Georgia cohort to apply the 1994 case definition using both the 1997 Wichita [18] and the 2004 Georgia [19] methods. The objectives of this analysis are to directly compare the extent of agreement in case ascertainment and to compare the illness characteristics of participants in GA-T1 classified as CFS by the two methods.

## Methods

### Data source and study sample

Data came from the follow-up of a population-based study of CFS in Georgia (GA-T1). The CDC Institutional Review Board approved the study and all participants provided informed consent.

**Fig. 1:**
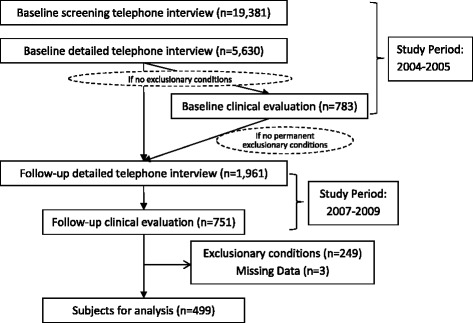
Schematic diagram for GA-T1 study conducted during 2007–2009

Details of the baseline study conducted in 2004–2005 have been published [19, 22]. In brief, a random-digit-dialing screening telephone interview identified respondents who were ill 6 months or longer with one or more of the CFS illness domains of fatigue, sleep, pain, or cognition, and those who were well. Eligible respondents were randomly selected for a detailed telephone interview to identify exclusionary conditions and features of CFS. After the detailed interview, all participants who appeared to meet criteria for CFS (CFS-like), non-ill controls (matched to CFS-like on age, sex, race, and residence), and randomly selected participants (number equal to the total of CFS-like and controls) from an intermediate group (ill but not meeting all CFS-like criteria) were invited for clinical evaluation. The clinical visit was completed by 783 persons and included detailed medical history, physical examination, laboratory tests, psychiatric evaluation, and questionnaires to measure functional impairment, fatigue, and other symptoms (Fig. 1).

Those eligible for the follow-up study (GA-T1) conducted during 2007–2009 (schematic diagram in Fig. 1) included the 3730 individuals from the phone-interviewed cohort in the baseline study (66 %) who did not have exclusionary conditions (i.e., all 681 participants seen in clinic without permanent exclusions and 3049 who only completed the baseline detailed telephone interview). Of those eligible, 1961 (53 %) completed the follow-up detailed telephone interview. Respondents invited to the follow-up clinical evaluation included all those eligible at baseline (including all seen in clinic who had no permanent exclusions) and newly identified CFS-like respondents along with well subjects matched on residence (metropolitan, urban, rural), sex, race/ethnicity, and age (within 3 years). Of the 1961 individuals who completed the follow-up detailed telephone interview, 919 (47 %) were eligible for the 1-day clinical evaluation and 751 (81 %) completed this evaluation.

The clinical evaluation included a detailed medical history, physical examination, laboratory tests, and the Structured Clinical Interview for DSM Disorders (SCID) to identify exclusionary medical and psychiatric conditions. All clinic participants completed the Zung self-rating depression scale (SDS) that includes 20 items measuring core symptoms of major depression during the past week [23]. Each item was scored on a Likert scale ranging from 1 to 4. A total score was derived by summing the individual item scores, and ranges from 20 to 80. A score >60 is considered moderate to severe depression. Of the 751 participants who completed the GA-T1 clinical evaluation, 249 (39 %) were identified as having one or more exclusionary medical and/or psychiatric conditions. Additionally, the exclusionary status of three individuals could not be determined due to incomplete lab results. These 252 individuals were removed from the current analysis, and the remaining 499 individuals without exclusionary conditions form the basis of this report.

### Methods of applying the 1994 CFS case definition

The 1994 CFS case definition specifies three major dimensions of CFS: fatigue, functional impairment, and eight accompanying symptoms (e.g., post-exertional malaise, impaired memory or concentration, sore throat, tender cervical or axillary lymph nodes, muscle pain, multi-joint pain, new headaches, unrefreshing sleep) [1]. Fatigue, functional impairment, and at least 4 of the eight symptoms need to be present for at least 6 months. We evaluated the same three dimensions on all study participants using two methods. Method 1 used direct questions to address each feature of the case definition as in the 1997 Wichita study [18]. Method 2 used questionnaires with the subscale thresholds used in the 2004 Georgia study (see Table 1) [19].

**Table 1:** Brief comparison of methods of applying the 1994 case definition of CFS (see text for details)

	Method 1 – 1997 Wichita method	Method 2 – 2004 Georgia method
Fatigue	Telephone interview – “Severe fatigue, extreme tiredness, or exhaustion” - Duration 6 months or longer “Yes” AND - Rest makes fatigue a lot better “No” OR “Yes” – Some of the time, a little of the time, or hardly ever	Duration 6 months or longer AND MFI-20 General Fatigue ≥13 OR MFI-20 Reduced Activity ≥10
Functional Impairment	Telephone interview – “Yes” to one: “Has this severe fatigue, extreme tiredness or exhaustion substantially limited … …your ability to do your usual job or occupation? …your ability to do your usual educational activities …substantially limited your social, leisure or recreational activities?”	SF-36 v2 Physical Function ≤70 OR Role Physical ≤50 OR Social Function ≤75 OR Role Emotional ≤66.7
4 of 8 Case Defining Symptoms^a^	During the past month how often have you had < SYMPTOM > − “All the time” OR “Most of the time” AND Was < SYMPTOM > bothering you 6 months or longer – “Yes”	CDC Symptom Inventory <SYMPTOM > frequency X intensity scores summed for 8 case defining symptoms ≥25 and scores for at least 4 symptoms

*Abbreviations: MFI-20* multidimensional fatigue inventory-20 questions, *SF-36* medical outcomes survey short form-36 questions

^a^CFS case defining symptoms: post-exertional malaise, impaired memory or concentration, sore throat, tender cervical or axillary lymph nodes, muscle pain, multi-joint pain, new headaches, unrefreshing sleep

#### Method 1*:* the 1997 Wichita method

##### Fatigue

≥ 6 months duration, not relieved by rest. During the detailed telephone interview, we asked participants questions about the occurrence, duration, and frequency of their fatigue (severe fatigue, extreme tiredness, or exhaustion). If participants responded “yes” to fatigue in the past month, they were asked whether they had this fatigue persisting or relapsing for 6 months or longer (response choices: “yes” or “no”). If yes, we asked whether rest made their fatigue a lot better (response choices: “yes” or “no”); participants responding “yes” were asked how often this fatigue was relieved by rest (response choices: “all of the time”, “most of the time”, “some of the time”, “a little of the time”, or “hardly ever”). Participants were considered to meet fatigue criterion if they reported fatigue persisting or relapsing for 6 months or longer and responded “no” to fatigue made a lot better by rest or fatigue relieved by rest “some of the time”, “a little of the time”, or “hardly ever”.

##### Functional impairment

All participants responding “yes” to severe fatigue, extreme tiredness, or exhaustion for 1 month or longer in the screening interview were questioned during the detailed telephone interview to determine functional impairment, defined as substantial reduction in pre-illness level of occupational, educational, social, or personal activities. Participants were asked about each area of functioning in three separate questions (response choices: “yes”, “no”, “don’t know”, “not applicable”, or “refused”). Participants were considered to meet the functional impairment criterion if they responded “yes” to any of the following three questions:

(i) “Has this severe fatigue, extreme tiredness, or exhaustion substantially limited your ability to do your usual job or occupation?”
(ii) “Has this severe fatigue, extreme tiredness, or exhaustion substantially limited your ability to do your usual educational activities?”
(iii) “Has this severe fatigue, extreme tiredness, or exhaustion substantially limited your social, leisure, or recreational activities?”

##### Case defining symptoms

We asked all participants about their experience during the past month with each of the eight symptoms specified in the 1994 case definition, e.g., “during the past month how often have you had a sore throat?” with response choices “all of the time,” “most of the time”, “some of the time”, “rarely”, or “never”. Those responding “all” or “most of the time” for any symptom were asked if the symptom was bothering them 6 months or longer (response choices: “yes”, “no”, “don’t know”, “not applicable”, or “refused”). A symptom that was present “all” or “most of the time” and “yes” ≥6 months duration was considered to be endorsed. Participants endorsing ≥4 symptoms were considered to meet the symptom criterion.

#### Method 2*:* the 2004 Georgia method

##### Fatigue

We assessed fatigue severity with the 20-item Multidimensional Fatigue Inventory (MFI-20) administered at the clinic [24, 25]. Higher scores in each MFI-20 subscale (range: 4 to 20) indicate more severe fatigue. Those scoring ≥13 in the General Fatigue subscale or ≥10 in the Reduced Activity subscale were considered to meet the fatigue criterion. As population normative values were not available at that time, cut-off was based on the median scores from the non-fatigue well group in the Wichita clinical study. Subsequently normative values for a German population were published. The selected subscale thresholds are higher than the 75th percentile for males and females age 40–59 (10 and 11, respectively for General Fatigue; 9 and 11, respectively for Reduced Activity subscale) [26].

##### Functional impairment

We assessed functional impairment using the Medical Outcomes Survey Short Form-36 version 2 (SF-36 v2) administered at the clinic [27, 28]. Lower SF-36 subscale scores indicate worse functioning (range: 0 to 100). The item responses for the subscales of Role Physical and Role Emotional were collapsed into yes/no for consistency with SF-36 v1 used in previous studies [19, 20]. Those scoring below the 25th percentile of published data for the 1998 US general population in any one of four subscales [Physical Function (≤70), Role Physical (≤50), Social Function (≤75), Role Emotional (≤66.7)] were considered to meet the functional impairment criterion.

##### Case defining symptoms

We assessed symptoms using the CDC Symptom Inventory (SI) administered at the clinic [29]. The questionnaire (provided in supplementary material) asks about the frequency and intensity of symptoms experienced during the past month (the eight CFS-defining symptoms, as well as an additional 11 illness symptoms). The revised version of the CDC SI used in this study provided a five-point scale for frequency and intensity of 19 symptoms. Participants were asked to report the frequency (1 = “a little of the time”, 2 = “some of the time”, 3 = “a good bit of the time”, 4 = “most of the time”, 5 = “all of the time”) and intensity or severity (1 = “very mild”, 2 = “mild”, 3 = “moderate”, 4 = “severe”, 5 = “very severe”). For consistency with the original version of CDC SI [30], we collapsed the responses into the following categories: the frequency response value (1 = “a little of the time”, 2 = “some of the time,” 3 = “a good bit of the time” or “most of the time”, 4 = “all of the time”) by the severity or intensity response value (1 = “very mild” or “mild”, 2.5 = “moderate”, 4 = “severe” or “very severe”). Symptoms that had been present less than 6 months were scored 0. For symptoms that had been present for at least 6 months, individual symptom scores were calculated by multiplying the frequency value by the intensity value. The CFS symptom summary score was calculated as the sum of the eight CFS individual symptom scores. Participants with at least four of the eight CFS symptoms for at least 6 months and a CFS symptom summary score ≥25 were considered to meet the symptom criterion [29].

### Statistical analysis

We calculated the concordance percentage and Kappa coefficient to examine the agreement between case ascertainment using each method of applying the 1994 CFS case definition: Method 1 (M1; 1997 Wichita) and Method 2 (M2; 2004 Georgia). We examined the fatigue, functional impairment, and symptom profiles of the CFS cases identified with both methods (M1/M2), compared to the CFS cases identified only with Method 2 (only M2) and the CFS cases identified only with the Method 1 (only M1). General linear models were used to examine the group effect and ad-hoc comparisons across three groups (M1/M2, only M1, and only M2) were also performed with Bonferroni correction for the *p*-value adjustment. All tests of significance were two-sided with the alpha level set at 0.05.

## Results

Table 2 summarizes the demographics of the study sample, as well as duration and onset of fatiguing illness for those classified as CFS. The majority were female (73.55 %), white (78.96 %), lived in a rural or urban area (81.36 %), or had at least some college education (80.16 %). The mean age of participants was 47.61 years. Of the 499 subjects without exclusionary conditions, 86 were classified as CFS by one or both methods: 59 with Method 1 (M1), and 71 with Method 2 (M2). There were no statistically significant differences in the proportion of females, mean duration of fatigue, or proportion with sudden onset among CFS cases classified by the two methods. As shown in Table 3, the Kappa statistic for agreement was substantial (0.63; 95 % CI: 0.53, 0.73) and the overall concordance of classification for the two methods was 91.59 %. There were 44 cases identified by both methods (M1/M2), 15 cases only identified by Method 1 (only M1), and 27 cases only identified by Method 2 (only M2).

**Table 2:** Characteristics of Study Sample (n=499)

	All Subjects (n=499)	Classified as CFS	
		1994 Case Definition Method	
		*Method 1* (n=59)	*Method 2* (n = 71)
Characteristics^a^ [values are n (%)			
Age in Yrs, Mean (SD)	47.61 (9.84)	46.76 (9.82)	47.93 (9.90)
Sex			
Female	367 (73.55)	49 (83.05)	65 (91.55)
Male	132 (26.45)	10 (16.95)	6 (8.45)
Race			
Black	98 (19.64)	8 (13.56)	12 (16.90)
White	394 (78.96)	48 (81.36)	56 (78.87)
All Others	7 (1.40)	3 (5.08)	3 (4.23)
Geographic Area			
Metropolitan	93 (18.64)	7 (11.86)	8 (11.27)
Urban	161 (32.26)	29 (49.15)	39 (54.93)
Rural	245 (49.10)	23 (38.98)	24 (33.80)
Married	351(70.62)	38 (64.41)	46(64.79)
Education			
≤ High School	99 (19.84)	15 (25.42)	17 (23.94)
Some College	165 (33.07)	22 (37.29)	27 (38.03)
≥ College	233 (46.69)	22 (37.29)	27 (38.03)
Type of onset			
Sudden	n/a	10 (16.95)	11 (15.49)
Gradual	n/a	46 (77.97)	36 (50.70)
Not known	n/a	3 (5.08)	24 (33.80)
Duration Fatigue in Yrs, Mean (SD)	n/a	12.13(8.92)	12.26 (9.87)

*n/a* not applicable

^a^Values are n (%) unless otherwise indicated

**Table 3:** Comparison of CFS Case Ascertainment using Two Methods of Applying the 1994 Case Definition (n=499)

		Method 1	
		CFS	Non-CFS
*Method 2*	CFS	44	27
	Non-CFS	15	413
	Kappa= 0.63, 95 % CI= 0.53 - 0.73.		
	Concordance= 91.59 %		
	Discordance= 8.41 %		

Table 4 shows the fatigue, functional impairment, and symptom profile as measured by MFI-20, SF-36 and CDC SI as well as depression scores for participants classified as CFS, divided into three non-overlapping groups (M1/M2, only M1, and only M2). Participants identified with both methods (M1/M2) were in general more fatigued, had more functional impairment, more CFS symptoms, higher symptom scores and higher Zung scores compared to those identified by only one method. Comparing characteristics of the CFS cases identified by only one of the two methods highlights differences that could be missed if comparisons of M1 and M2 included those identified by both methods. There were no statistically significant differences between the groups identified only by Method 1 (only M1) and only by Method 2 (only M2) on the MFI-20 subscales except for the General Fatigue subscale, where the only M2 group was more severe (difference of 2.39 points). In four of the eight SF-36 subscales, individuals in the only M2 group had significantly more functional impairment than the only M1 group (Physical Functioning, Role Physical, Social Functioning, and Bodily Pain; range of differences 13.53 to 29.00). Those in the only M2 group also had significantly higher symptom scores than the only M1 group (score difference 17.12). Mean SF-36 scores for Vitality and Mental Health were not significantly different between the only M1 and only M2 groups, but the M1/M2 group scores in Vitality and Mental Health (20.80, 55.55) were significantly lower than the only M1 group (36.33, 75.73), indicating more severe impairment. While not statistically significant, the proportion of individuals who experienced post-exertional malaise 6 months or longer was less in the only M1 group (73.33 %) than in M1/M2 (81.82 %) or only M2 (77.8 %) groups. Only one CFS case had moderate to severe depression (Zung score >60), and both methods classified this participant as CFS.

**Table 4:** Comparison of CFS Cases identified with Two Methods of Applying the 1994 Case Definition

	CFS Classification (non-overlapping categories)		
	Both Methods	*Method 1* Only	*Method 2* Only
	(n=44; M1/M2)	(n=15, only M1)	(n=27; only M2)
MFI-20			
**General Fatigue** ^b, c^	17.05 (0.35)	14.20 (0.89)	16.59 (0.46)
**Reduced Activity** ^a^	12.95 (0.66)	10.27 (1.02)	10.56 (0.66)
Physical Fatigue ^a, b^	14.89 (0.41)	11.67 (1.04)	12.19 (0.70)
Mental Fatigue	13.36 (0.67)	12.47 (1.18)	11.33 (0.69)
Reduced Motivation	12.84 (0.56)	10.47 (0.94)	11.56 (0.65)
SF-36			
**Physical Functioning** ^c^	58.64 (3.47)	78.67 (4.77)	64.81 (5.14)
**Role Physical** ^c^	31.25 (5.92)	71.67 (7.66)	42.59 (7.76)
**Social Functioning** ^c^	45.17 (3.28)	70.83 (5.41)	55.09 (3.96)
**Role Emotional**	46.21 (6.52)	75.55 (8.89)	70.37 (7.82)
Bodily Pain ^a, b, c^	35.20 (2.38)	60.20 (4.58)	46.67 (3.37)
General Health ^a, b^	39.59 (2.63)	66.00 (4.58)	52.48 (3.52)
Vitality ^b^	20.80 (1.93)	36.33 (4.59)	27.41 (3.37)
Mental Health ^b^	55.55 (3.26)	75.73 (4.56)	65.19 (3.83)
CDC SI			
**Number of CFS Symptoms** ^a, b^	5.55 (0.19)	4.6 (0.19)	4.74 (0.13)
**CFS Symptom Summary Score** ^a, b, c^	48.84 (2.22)	23.23 (2.02)	40.35 (1.94)
PEM in CDC SI^d^, n (%)	36 (81.82%)	11 (73.33%)	21 (77.78%)
Depression			
Zung SDS Score^b^	46.48 (1.17)	37.20 (2.02)	42.00 (1.35)
Depression (mod – severe), n (%)	1 (2.27%)	0	0

Sample mean was listed for each group and standard error of mean (SEM) was listed in parenthesis unless otherwise noted. Bonferroni correction was used for the p-value adjustment for the multiple group comparison

Bold font indicates the subscales used by *Method 2* in establishing criteria for 1994 Case Definition

^a^ Significant difference between M1/M2 and only M2

^b^ Significant difference between M1/M2 and only M1

^c^ Significant difference between only M1 and only M2

^d^ PEM = Score ≥ 7.5 for post-exertional fatigue symptom in CDC SI

Table 5 summarizes the percentages of CFS cases in each of the three groups that met each of the MFI-20, SF-36 and Symptom Inventory thresholds used in Method 2. For the fatigue criterion, a higher percentage of those in the M1/M2 and only M2 CFS groups met the General Fatigue cutoff than those in the only M1 group (95.45, 96.3 and 60 % respectively). A linear decreasing trend was observed in the percentage of individuals meeting the Physical Functioning cutoff, with the only M1 group having the lowest percentage of all three groups (72.73, 51.85, and 20 %, respectively). The only M1 group also had a much smaller percentage of individuals meeting the cutoff for Role Physical and Social Functioning. The proportion of those meeting the Role Emotional criterion did not differ between only M1 and only M2 groups. No instance in which the SF-36 criterion was fulfilled by meeting only the Role Emotional cutoff was observed in any group. Only 33.3 % of the only M1 group had CFS symptom summary score of ≥25, whereas this was a requirement for M2, so 100 % of those in M1/M2 and only M2 groups met this criterion. Memory or concentration problems lasting for six months or longer were less frequent in individuals in the only M2 group (25.93 %), compared to those in M1/M2 (56.82 %) and only M1 (46.67 %) groups.

**Table 5:** MFI, SF-36 and Symptom Inventory Criteria for CFS - Comparison of CFS Cases identified with Two Methods of Applying the 1994 Case Definition

	CFS Classification (non-overlapping categories)		
Criteria	Both Methods	*Method 1* Only	*Method 2* Only
	(n=44; M1/M2)	(n=15; only M1)	(n=27; only M2)
(1) MFI Criterion (one of two cutoffs)			
(i) General Fatigue ≥ 13*	42 (95.45 %)	9 (60.00 %)	26 (96.30 %)
(ii) Reduced Activity ≥ 10	34 (77.27 %)	7 (46.67 %)	18 (66.67 %)
Met at least one*	44 (100 %)	11 (73.33 %)	27 (100 %)
(2) SF-36 Criterion (one of four cutoffs)			
(iii) Physical Functioning ≤ 70*	32 (72.73 %)	3 (20.00 %)	14 (51.85 %)
(iv) Role Physical ≤ 50*	33 (75.00 %)	4 (26.67 %)	19 (70.37 %)
(v) Social Functioning ≤ 75*	42 (95.45 %)	11 (73.33 %)	25 (92.59 %)
(vi) Role Emotional ≤ 66.67	29 (65.91 %)	6 (40.00 %)	11 (40.74 %)
Met at least one*	44 (100 %)	11 (73.33 %)	27 (100 %)
(3) CDC Symptom Criterion (Both cutoffs)			
(vii) ≥ 4 CFS Symptoms lasting ≥ 6 months	44 (100 %)	15 (100 %)	27 (100 %)
(viii) Summary Score ≥ 25*	44 (100 %)	5 (33.33 %)	27 (100 %)
Met both	44 (100 %)	5 (33.33 %)	27 (100 %)
Meet all criteria (1),(2), (3)	44 (100 %)	0	27 (100 %)
*Meets SF-36 Criterion based on Role Emotional Alone*	0	0	0
Total number of criteria (i)-(viii) met (8 maximum)			
1		1 (6.67 %)	
2		1 (6.67 %)	
3		3 (20.00 %)	
4	2 (4.55 %)	5 (33.33 %)	1 (3.70 %)
5	7 (15.91 %)	3 (20.00 %)	7 (25.93 %)
6	4 (9.09 %)	1 (6.67 %)	8 (29.63 %)
7	15 (34.09 %)	1 (6.67 %)	8 (29.63 %)
8	16 (36.36 %)	0	3 (11.11 %)

* indicates p-values < 0.05 for χ^2^ testing of the independence across three groups

Differences between the groups were also highlighted by the total number of MFI-20, SF-36 and symptom criteria that were met, eight maximum (Table 5). Surprisingly, only 33 % of those in the only M1 group had four or more case defining symptoms above threshold in SI score. Although there were participants in M1/M2 and only M2 groups that met all eight scoring cutoffs (36.36 and 11.11 %, respectively), none in the only M1 group met all eight.

## Discussion

The present study indicates that even when using the same case definition and the same study sample, methods of applying the case definition can impact CFS classification. We applied the 1994 case definition of CFS using direct questions to address case definition criteria (Method 1) as well as a method based on the use of questionnaires with subscale score thresholds for each dimension (Method 2). While some differences were noted, classification based on each method showed substantial agreement (kappa = 0.63 and overall concordance 91.6 %).

Method 2 identified more participants as meeting the 1994 case definition of CFS than did Method 1 (71 compared with 59). This could occur if Method 2 is less specific or more sensitive than Method 1. In the absence of a gold standard for true classification, standardized instruments measuring fatigue (MFI-20), function (SF-36), and symptom frequency and severity (CDC-SI) allow direct comparison of participants classified by either method alone or by both methods. Differences between the methods are highlighted by comparing these measures in participants classified as CFS by only one method. Individuals classified as CFS only by Method 2 (only M2 group) have significantly higher scores in General Fatigue, significantly lower scores in Physical Functioning, Role Physical, and Social Functioning, and significantly higher symptom summary score than those only identified with Method 1 (only M1). In all other MFI-20, SF-36, and CDC SI measures the two groups did not differ. This provides assurance that Method 2 does not identify a population that is less severely affected. Individuals classified as CFS by both methods (M1/M2 group) were in general more fatigued, had more functional impairment, more CFS symptoms, and higher CFS symptom summary scores compared to those in the only M1 or only M2 groups. Additionally, Method 2 includes the threshold of 25 for the CFS symptom summary scores, enabling better quantifying of CFS symptom severity as a whole. About 67 % of those identified only with Method 1 failed to exceed this symptom severity threshold.

Inclusion of the SF-36 Role Emotional score as one option to meet the functional impairment criterion has led to criticism that Method 2 could identify individuals with major depressive disorder (MDD) rather than CFS [21]. In fact, none of those identified as CFS by any method met the functional impairment criterion based only on Role Emotional (Table 5). Only one CFS patient (identified by both methods) had moderate to severe depression (Zung ≥60). All three groups had higher mental health SF-36 scores (55.55 for M1/M2, 65.19 for only M2, and 75.73 for only M1) and role emotional functioning (46.21 for M1/M2, 70.37 for only M2, and 75.55 for only M1) than the SF-36 norms observed among patients with depression in outpatient clinics (36.37 and 38.60, respectively; [28]). However, the mean scores for Vitality (an estimate of vitality, energy, and fatigue) for those classified by both methods (20.80) and those by Method 2 only (27.41) were much lower than the mean score reported for depressive patients (39.91) [28].

While the present study focuses on the use of questionnaires to establish minimal criteria for case ascertainment in a population-based surveillance study, it is clear that the instruments provide additional value by providing measures of illness that can be used to stratify or subgroup CFS. The questionnaires could serve as patient/person reported outcome measures to describe the natural history of the illness, identify and quantify change in response to interventions, and provide criteria to identify patient populations with similar characteristics for basic research and clinical trials. As shown by the above comparisons, these instruments allow direct comparison of illness severity between groups, as well as between persons with CFS and other illnesses. Further studies designed to establish the reliability and validity of each of these instruments for CFS and other illnesses, as well as to identify clinically meaningful changes, are needed.

The study design included rigorous screening including clinical, laboratory, and psychological evaluation (including SCID) to identify exclusionary medical and psychiatric conditions. In the absence of this screening process, both methods of applying the 1994 case definition could identify a very different sample. Failure to screen for exclusionary conditions prior to using the Method 2 algorithm may explain the perception that it generates significant classification errors [21].

While the current analysis provides reassurance that the 10-fold difference in the population- based prevalence estimates in the 1997 Wichita and 2004 Georgia studies [18, 19] is not due to inclusion of those with psychiatric illnesses or those less severely affected, it does not explain the prevalence difference. The increased detection with Method 2 compared to Method 1 does not fully explain the difference in prevalence. Other differences in study design, such as the initial household screening based on fatigue, pain, cognition, and sleep rather than restricting to fatigue, restricted age of enrollment (18–59 years), match criteria, and weighting of estimates undoubtedly contributed to the different prevalence estimates between the two studies. The Georgia study identified more participants as eligible for clinic evaluation because fewer exclusions were based on information provided in the telephone interviews.

Further work needs to be done to evaluate the impact of using different cut-off values for each criterion as well as using other standardized instruments to measure the illness domains of CFS. Instruments that have been rigorously validated in general populations and therefore have established general population norms, such as the SF-36 and MFI-20, may not capture all aspects of function and fatigue in CFS. While one study did evaluate MFI-20 in CFS [30], a recent review of available measures of CFS indicates that few have been used in more than one study and evaluation of instrument performance needs improvement [31]. In addition, using instruments such as those developed by the NIH Patient Reported Outcomes Measurement Information System (PROMIS) initiative (http://www.nihpromis.org/about/overview) for application in a wide variety of chronic diseases and conditions will allow direct comparison of CFS to other conditions.

Changes in the CFS case definition would require different algorithms and different approaches to operationalize them for research. Most definitions recognize the same domains of illness but vary in the number of required symptoms. Another advantage of using standardized instruments to operationalize the case definition for research studies is that participants could be reclassified and stratified depending on the needs of the study.

## Conclusions

Even when using the same CFS case definition, methods of applying the case definition influence case ascertainment with subsequent impact on observed disease prevalence and severity. Research studies of CFS patients need to specify both the case definition and the specific approach and tools or instruments used to apply the definition. Use of standardized instruments for the major domains of CFS such as fatigue (MFI-20), functional impairment (SF-36), and symptoms (CDC-SI) has advantages for ascertainment, disease stratification, and comparing CFS patients to other illnesses. The IOM report on ME/CFS recommended clinical evaluation of these and other standardized instruments. Further studies are needed to optimize thresholds for criterion identification and to validate clinically meaningful changes in scores.
